# A cross-tissue transcriptome association study identifies key genes in essential hypertension

**DOI:** 10.3389/fgene.2023.1114174

**Published:** 2023-02-10

**Authors:** Sihui Huang, Jie Wang, Nannan Liu, Ping Li, Sha Wu, Luming Qi, Lina Xia

**Affiliations:** ^1^ College of Health and Rehabilitation, Chengdu University of Traditional Chinese Medicine, Chengdu, China; ^2^ Key Laboratory of Traditional Chinese Medicine Regimen and Health Industry Development, State Administration of TCM, Chengdu, China; ^3^ Leshan Vocational and Technical College, Leshan, China

**Keywords:** hypertension, post-gwas, TWAS, SMR, UTMOST

## Abstract

Genome-wide association study (GWAS) have identified over 1,000 loci associated with blood pressure. However, these loci only explain 6% of heritability. Transcriptome-wide association studies (TWAS) combine GWAS summary data with expression quantitative trait loci (eQTL) to provide a better approach to finding genes associated with complex traits. GWAS summary data (N = 450,584) for essential hypertension originating from European samples were subjected to Post-GWAS analysis using FUMA software and then combined with eQTL data from Genotype-Tissues Expression Project (GTEx) v8 for TWAS analysis using UTMOST, FUSION software, and then validated the results with SMR. FUMA identified 346 significant genes associated with hypertension, FUSION identified 461, and UTMOST cross-tissue analysis identified 34, of which 5 were common. SMR validation identified 3 key genes: *ENPEP*, *USP38,* and *KCNK3*. In previous GWAS studies on blood pressure regulation, the association of *ENPEP* and *KCNK3* with hypertension has been established, and the association between *USP38* and blood pressure regulation still needs further validation.

## 1 Introduction

Hypertension is an important risk factor for many diseases, such as dementia, chronic kidney disease, ischemic heart disease, stroke, and other cardiovascular diseases (CVD) (Zhou et al., 2021). From 1990 to 2019, the number of people aged 30 to 79 with hypertension worldwide increased from 650 million to 1.28 billion (NCD Risk Factor Collaboration, 2021). Between 1990 and 2015, as the rate of elevated systolic pressure increased, so did the disabilities and deaths associated with it (Forouzanfar et al., 2017). Elevated blood pressure contributes to a huge global burden of CVD and premature death. Essential hypertension is a multi-gene and multi-factor disease caused by the interaction of environment and heredity and it accounts for about 95% of hypertension cases.

The heritability of blood pressure is estimated to be 30%–50% (Russo et al., 2018), and twin studies showed that monozygotic twins were more correlated with blood pressure phenotypes than dizygotic twins (Luft, 2001). A multi-generational cohort study indicated that early-onset hypertension in grandparents increased the risk of high blood pressure in grandchildren (Niiranen et al., 2017). However, the first genome-wide association study (GWAS) for hypertension can’t find significant loci (Hirschhorn and Daly, 2005), possibly because hypertension has fewer common risk alleles but has a large effect size that was not detected due to the limitations of genetic analysis and analytical methods (Wellcome Trust Case Control, 2007). Two years later, a GWAS meta-analysis based on six cohorts identified 10 SNPs associated with hypertension and mapped them to a corresponding gene *ATP2B1* (Levy et al., 2009). In a subsequent meta-analysis of blood pressure in more than one million people, 535 novel loci associated with blood pressure were identified (Evangelou et al., 2018). The GWAS research has continued for over a decade, and it has found over 1,000 blood pressure loci which explain about 6% of the heritability based on single nucleotide polymorphism (SNP) (Olczak et al., 2021). However, most of the disease trait loci identified by GWAS studies are located in non-coding regions, so it’s difficult to evaluate their function (Maurano et al., 2012). Transcriptome-wide association study (TWAS) integrates individual-level genotype data or GWAS summary data with expression quantitative trait loci (eQTL) to evaluate the association between gene expression level and complex traits or diseases (Page et al., 2021). Compared to GWAS, TWAS is a gene-based association approach with tissue specificity, and it has a lower burden of multiple testing (Page et al., 2021). In recent years, TWAS is widely used in genetic epidemiology to search for genes associated with complex phenotypes (Dall'Aglio et al., 2021; Pairo-Castineira et al., 2021).

To find the key genes of essential hypertension, we integrated the GWAS data including 450,584 samples and the eQTL file from Genotype-Tissues Expression Project (GTEx) v8 (Consortium, 2020), which were analyzed using FUMA (Watanabe et al., 2017), UTMOST (Hu et al., 2019), and FUSION (Gusev et al., 2016) software respectively, and we finally validated the results using SMR (Zhu et al., 2016). The main target organs of hypertension are the heart, kidneys, brain, retina, and arteries, and the activation of the renin-angiotensin-aldosterone system, oxidative stress, and inflammation plays an important role in the pathogenesis of hypertension (Kucmierz et al., 2021). Therefore, hypertension may hurt various systems within the body. We used the eQTL information of all the tissues and finally identified three key genes associated with essential hypertension: *ENPEP*, *USP38,* and *KCNK3*.

## 2 Materials and methods

### 2.1 Datasets

To obtain a larger sample size, we identified hypertension GWAS summary data from two databases and performed a meta-analysis using metal software (http://csg.sph.umich.edu/abecasis/metal/) to get statistics of greater efficacy. One contains a sample of 244,890 participants (71,332 cases, 173,558 controls) from the UK Biobank by Kyoko Watanabe (https://atlas.ctglab.nl) and the other 205,694 participants (42,857 cases, 162,837 controls) from FinnGen (https://www.finngen.fi/en/access_results). Based on the *p*-values of the two GWAS summary data SNPs, we used a fixed effects inverse variance weighted meta-analysis in METAL software (Willer et al., 2010). The direction of effect and *p*-value in each study were converted into Z-score, and the Z-score for each allele were combined in a weighted form in the sample with weights proportional to the square root of the sample size in each study (Willer et al., 2010). To improve statistical validity, we screened the common SNPs in two GWAS datasets with a total of 8,436,487. The main model weights file of gene expression was derived from GTEx v8 (16). The reference panel used to estimate linkage disequilibrium (LD) was derived from the 1,000 Genomes Europe sample (Genomes Project et al., 2015).

### 2.2 Characterization and annotation of genetic associations with functional mapping and annotation

Functional Mapping and Annotation (FUMA) serves as a post-GWAS analysis tool, integrating information from multiple biological resources to perform gene prioritization, functional annotation, and visual analysis of GWAS results (Watanabe et al., 2017). We performed the following analysis of the hypertension GWAS data prepared in advance using FUMA (v1.3.7). First, appropriate parameters for determining the priority genes were set. Independent significant SNPs were defined as genome-wide *p* < 5 × 10^−8^ and LD *r* ^*2*^ < 0.6. Lead SNPs were defined as those independent from each other at *r* ^*2*^ < 0.1 inside a subset of independent significant SNPs (Watanabe et al., 2017). Based on the lead SNPs, genomic risk loci were physically located close to or overlapped with these independent signals. The distance of the genetic risk region was determined according to the default of 250 kb. The data from phase 3 of the European 1000G project were used for the LD reference.

Next, gene analysis and gene-set analysis were performed using the default parameters of the MAGMA (v1.08) software implemented in FUMA. The gene analysis with MAGMA is based on multilinear principal component regression, where the SNP matrix of genes is projected onto the principal components and the F-test is used to calculate the gene *p*-values (de Leeuw et al., 2015). Therefore, a gene analysis on input GWAS data can be done in MAGMA. If SNPs are located within the gene, the gene-base *p*-value of the protein-coding gene is calculated by mapping the SNPs to the gene (Watanabe et al., 2017). The threshold for Bonferroni correction is 0.05/18,800 (number of genes tested). The gene-set analysis is divided into two distinct and largely independent parts: self-contained and competitive gene-set analysis, so it is more flexible (de Leeuw et al., 2015). The gene-set analysis used 10,678 gene sets from MsigDB v6.2, with 4,761 curated gene sets and 5,917 GO terms. Bonferroni correction was performed for all tested gene sets.

### 2.3 Transcriptome-wide associationstudies

#### 2.3.1 Transcriptome-wide association studies analysis with UTMOST

Because hypertension is associated with all systems of the body (Kucmierz et al., 2021), the conventional approach to TWAS analysis is to train separate attribution models for different tissues (Gusev et al., 2016). Such hypothesis-free searches across genes and tissues increase the burden of multiple testing and reduce statistical power (Hu et al., 2019). However, a new method Unified Test for Molecular SignaTures (UTMOST) (http://zhaocenter.org/UTMOST), which combines multiple single-tissue associations into a single powerful metric to quantify the entire genetic-trait associations, can improve the accuracy of imputation (Hu et al., 2019). Single-tissue association tests were then performed in conjunction with the hypertension GWAS summary statistics. Finally, we used the generalized Berk-Jones (GBJ) test to combine single-tissue gene-trait associations. Considering the number of valid tests, we set the transcriptome-wide significance threshold for the joint test at *p* < 2.26193 × 10^−6^ (0.05/22,105).

#### 2.3.2 Transcriptome-wide association studies analysis with FUSION

Functional Summary-based Imputation (FUSION) was commonly used for TWAS analysis to estimate heritability, build predictive models, and determine transcriptome-wide associations (Li et al., 2021). We performed TWAS analysis with the above meta-merged hypertension GWAS data using the FUSION software with default settings (http://gusevlab.org/projects/fusion/). Then, we downloaded 49 tissues’ eQTL reference panels from GTEx v8 for which predictive models had been built.

Next, 1,000 Genomes European samples (https://data.broadinstitute.org/alkesgroup/FUSION/LDREF.tar.bz2) were used to estimate the LD between the prediction model and the SNP at each locus of GWAS (Gusev et al., 2019). The Bonferroni-corrected TWAS threshold was defined as *p* < 1.51 × 10^−7^ (0.05/330,952) (total number of predictive models).

### 2.4 Joint/conditional analysis

To distinguish whether multiple significant genes in a locus were independently associated or interlinked with each other, we performed joint/conditional tests using the post-process module in FUSION(25). Multiple associated genes within the region identified by TWAS were modeled jointly to determine which was the independent association signal, and each hypertension GWAS association was conditioned on the joint model, with one SNP at a time (Liao et al., 2019). After testing, genes that represent independent associations were termed jointly significant, and those that were no longer significant were termed marginally significant.

### 2.5 Bayesian colocalization

To determine whether the GWAS summary data and eQTL signal shared the same causal variant, we performed a Bayesian colocalization analysis using the COLOC R package (https://cran.r-project.org/web/packages/coloc/, version 5.1.0). The colocation test is based on sample size and converts the relevant statistics into an effect size. This approach estimates the posterior probability (PP) that two outcomes within a locus are associated and driven by a common causal variable (Dall'Aglio et al., 2021). There are 5 PP statistics for the hypothesis: H0 (not related), H1 (a causal variant for GWAS only), H2 (a causal variant for eQTL only), H3 (two independent causal variants), and H4 (a shared causal variant). If PP4 is larger (PP4 >75%), it indicates that a single variant affects both traits (Giambartolomei et al., 2014).

### 2.6 Results validation using SMR

A research method SMR based on Mendelian randomization was proposed by Zhu et al., in 2016 (20). It is a joint analysis of GWAS signal and expression and methylation quantitative traits loci (eQTL and mQTL, respectively) to determine the associations between gene expression levels and traits (Baranova et al., 2021). We analyzed the hypertension GWAS data using the eQTL data of 49 human tissues from GTEx v8 (n = 73–670) (Consortium, 2020). Genotype data from the European 1000 Genomes Project Phase 3 were used to estimate the LD. The heterogeneity in dependent instruments (HEIDI) test was conducted to estimate the heterogeneity between effects. We performed this analysis using the default parameters of the SMR software (https://yanglab.westlake.edu.cn/software/smr/#SMR). A strict Bonferroni-corrected SMR threshold of *p* < 0.05/number of probes, and *p* > 0.05 was used as the threshold for the HEIDI test (Restuadi et al., 2022).

## 3 Results

### 3.1 Study overview

**FIGURE 1:**
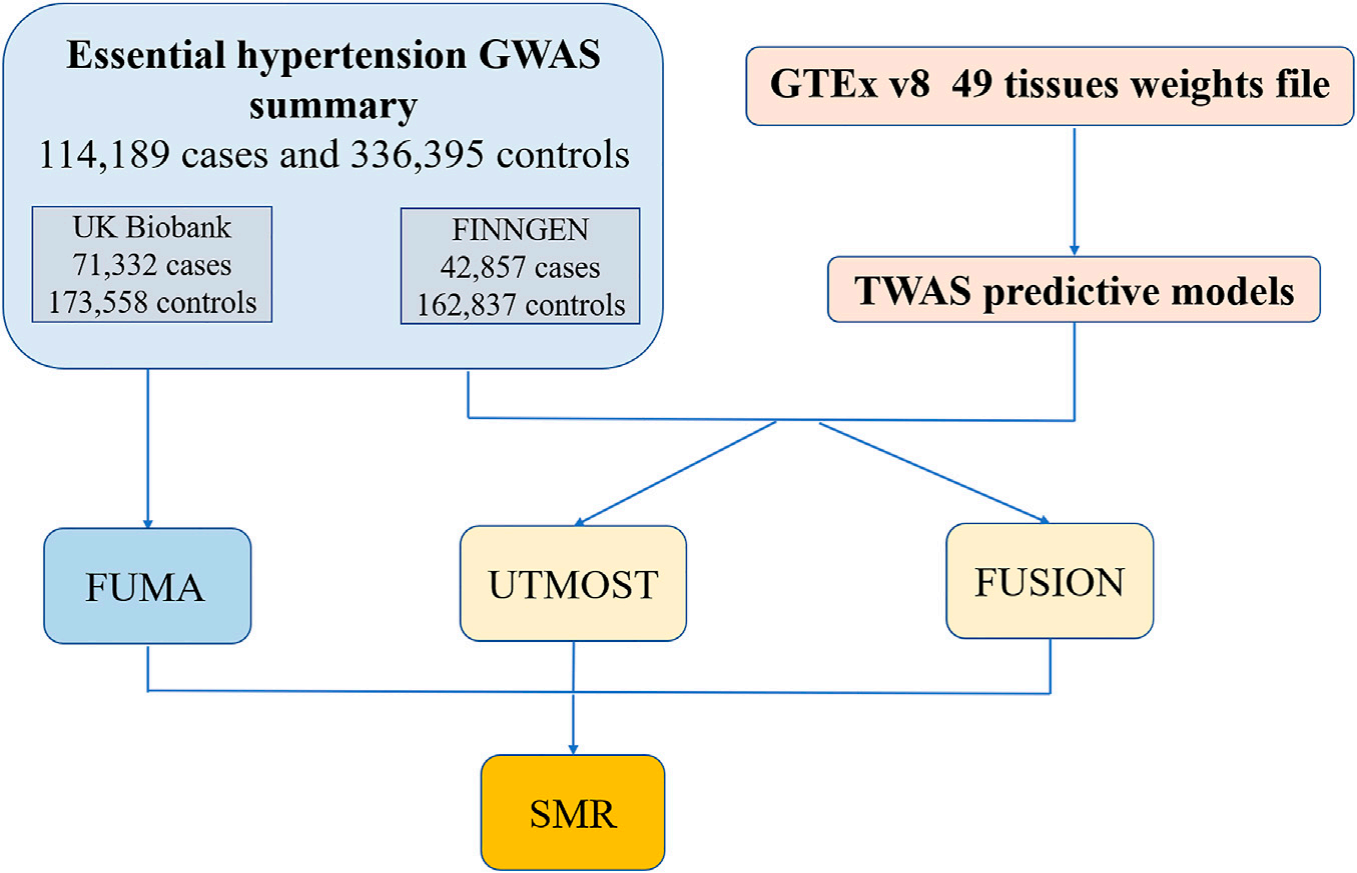
The workflow of the study. GWAS, genome-wide association; GTEx, Genotype-Tissues Expression Project; TWAS, transcriptome-wide association studies; FUMA, functional mapping and annotation; UTMOST, unified test for molecular signatures; FUSION, functional summary-based imputation; SMR, summary-data-based Mendelian randomization.

We used GWAS summary statistics regarding essential hypertension from two European sample databases to first perform Post-GWAS analysis on the FUMA platform to identify independently significant SNPs and corresponding genes. Next, we combined multiple tissues’ eQTL files from GTEx v8 and GWAS data for TWAS analysis. We used both the UTMOST and the FUSION software for TWAS analysis. The results of TWAS were merged with the genes identified by FUMA. Finally, the merged results were validated by SMR (Figure 1).

### 3.2 The results of post-genome-wide association study annotation by functional mapping and annotation

We used the FUMA platform to functionally annotate summary statistics of hypertensive GWASs, including 114,189 cases and 336,395 controls. Based on the previously set parameters, FUMA identified 173 genomic risk loci associated with essential hypertension (Supplementary Table S1). Within these loci, there were 248 lead SNPs and 662 independent significant SNPs (Supplementary Tables S2, S3). There were 21,127 candidate SNPs that had LD with the lead SNPs. Among the candidate SNPs, those with significant differences were located in intronic (10,958), intergenic (6,411), non-coding RNA intronic (2,278), UTR3 (271), and UTR5 (104) (Supplementary Table S4). Of the total number of SNPs, 7,848 were reported in previous studies, 27 of which were identified in one of the largest GWAS studies on hypertension by Evangelou et al., 2018.

**FIGURE 2:**
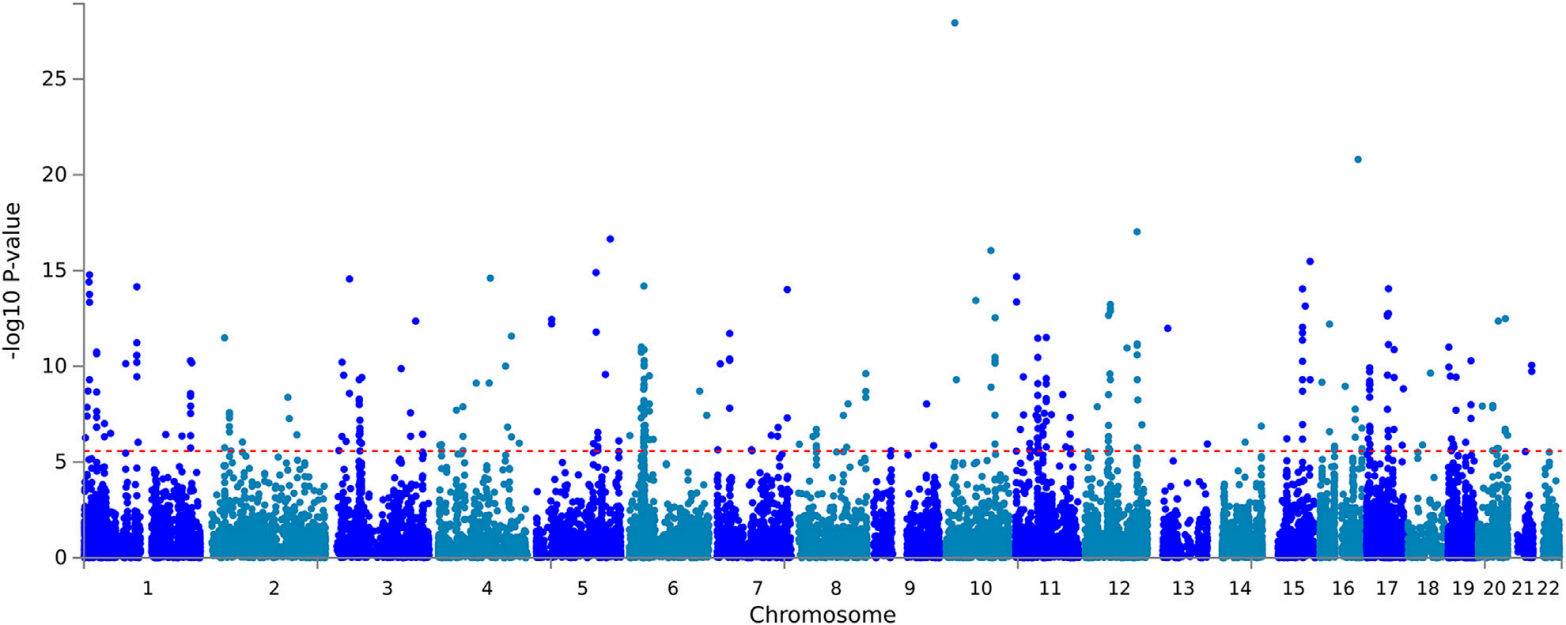
Manhattan Plot of MAGMA gene-based test. Gene-based testing using MAGMA based on input GWAS summary statistics for essential hypertension. Red lines indicate levels of genome-wide significance (-log_10_ *P*).

The input SNPs mapped to 18,800 protein-coding genes. According to the set threshold, 346 genes met the genome-wide significant requirement (Supplementary Table S5). The top three differentially expressed genes were *CACNB2* (*p* = 1.18E - 28), *CMIP* (*p* = 1.57E - 21) and *CUX2* (*p* = 9.47E - 18). The *CACNB2* gene has been reported in a previous genome-wide association study of blood pressure and hypertension (Levy et al., 2009). Based on the input GWAS summary statistics, significant genes identified by the MAGMA gene-based test were plotted on a Manhattan map (Figure 2). MAGMA’s gene-set analysis tested 15,483 gene sets using the default competitive testing model, with the top 10 gene sets provided in Supplementary Table S6. The top three most significant pathways were positive regulation of gene expression (*p* = 7.37E - 07), positive regulation of biosynthetic process (*p* = 1.22E - 06), and positive regulation of RNA biosynthetic process (*p* = 1.50E - 06). It is suggested that hypertension is associated with gene expression, RNA biological processes, which explains the heritability of hypertension.

### 3.3 Transcriptome-wide association studies result

#### 3.3.1 UTMOST single-tissue analysis

UTMOST single tissue analysis identified 483 significant gene-trait associations (Supplementary Table S7) between 83 genes and 49 tissues (49 tissues from GTEx v8). Of the 83 TWAS-significant genes, 15 had significant associations with more than ten tissues, including *KCNK3*, *SH3D19*, *AGBL5-IT1*, *ASXL2*, *BMP3*, *DPYSL5*, *USP38*, *ENPEP*, *RP11-286E11.2*, *LEF1*, *RAB10*, *TCF23*, *SNORA70F*, *MLPH*, and *TMEM214*. These 15 genes together contributed 310 (64.2%) of the 483 significant loci, suggesting that they are crucial in essential hypertension. Of these 483 significant loci, there were 203 significant loci in the heart, brain, kidney, and arterial tissues which are common targets of hypertension. There were 15 loci in the ovary, 14 loci in adipose subcutaneous, and 14 loci in the minor salivary gland, and the associations between these tissues and hypertension need further validation.

#### 3.3.2 UTMOST cross-tissue analysis

In the cross-tissue analysis, we found 34 genes that showed statistical significance, as detailed in Table 1. These 34 significant genes were all concentrated on three chromosomes:2, 4, and 18. Of the 15 significant genes identified in the previous single-tissue analysis, 14 were among the significant genes identified in the cross-tissue analysis. The remaining one, *MLPH*, is a gene encoding melanin, and no association with hypertension has been found in the previous literatures. Among the 34 significant genes identified by the cross-tissue analysis, *MFSD10* had no significant *p*-value in single-tissue analysis. The protein encoded by *MFSD10* may be involved in the efflux of organic anions. *MFSD10* has been identified as an eosinophil-specific surface molecule that may be an effective therapeutic target for eosinophil-related diseases (Nishimura et al., 2014). Whether there is an association between MFSD10 and hypertension needs further study.

**TABLE 1:** UTMOST cross tissue analysis result.

Gene symbole	Chromosomes	Ensemeble ID	Location (hg38)	Test_score	*p*_value
ENPEP	4	ENSG00000138792	110,365,733–110,565,285	27.18976656	5.13E-13
USP38	4	ENSG00000170185	143,184,917–143,224,429	24.91014084	5.63E-12
TCF23	2	ENSG00000163792	27,371,872–27,379,842	197.4985319	1.09E-11
BMP3	4	ENSG00000152785	81,030,708–81,057,627	383.2961589	2.00E-11
GCKR	2	ENSG00000084734	27,496,839–27,523,689	23.89284155	2.25E-11
KCNK3	2	ENSG00000171303	26,692,722–26,733,420	625.9908495	2.35E-11
DNAJC5G	2	ENSG00000163793	27,275,427–27,281,499	24.00199517	2.39E-11
GYPA	4	ENSG00000170180	144,109,303–144,140,854	24.45908507	2.48E-11
ASXL2	2	ENSG00000143970	25,733,753–25,878,487	299.3557665	2.55E-11
LEF1	4	ENSG00000138795	108,047,545–108,168,956	23.83871454	3.03E-11
SNORA70F	2	ENSG00000206869	164,687,643–164,687,777	180.3011514	5.48E-11
DPYSL5	2	ENSG00000157851	26,847,747–26,950,351	21.19784845	1.45E-10
AC019181.3	2	ENSG00000224331	164,687,287–164,687,596	21.8684064	3.45E-10
GRB14	2	ENSG00000115290	164,492,417–164,622,959	20.34623605	5.56E-10
FIGN	2	ENSG00000182263	164,459,121–164,592,518	17.28621027	6.21E-09
RAB10	2	ENSG00000084733	26,034,084–26,137,454	195.6345516	1.31E-08
RP11-286E11.2	4	ENSG00000249604	107,936,030–107,941,255	17.31587565	1.41E-08
MRFAP1	4	ENSG00000179010	6,640,091–6,642,729	17.47697332	1.53E-08
CENPA	2	ENSG00000115163	27,008,924–27,017,457	16.76721096	3.38E-08
AGBL5	2	ENSG00000084693	27,042,364–27,070,622	16.37489454	3.57E-08
PCDH18	4	ENSG00000189184	137,518,918–137,532,530	17.0542622	4.34E-08
C2orf16	2	ENSG00000221843	27,537,386–27,582,722	16.65592231	5.53E-08
MAPK4	18	ENSG00000141639	48,086,457–48,258,196	16.16287227	5.71E-08
SH3D19	4	ENSG00000109686	151,102,751–151,325,605	15.32021917	1.16E-07
TMEM214	2	ENSG00000119777	27,032,910–27,041,694	15.89230175	1.17E-07
MFSD10	4	ENSG00000109736	2,930,556–2,934,854	14.99286603	1.94E-07
LINC01630	18	ENSG00000227115	51,346,248–51,643,939	14.73942194	2.30E-07
ADGRF3	2	ENSG00000173567	26,308,173–26,346,817	13.55585128	4.71E-07
AGBL5-IT1	2	ENSG00000229122	27,061,037–27,061,815	280.6406527	8.90E-07
SCN3A	2	ENSG00000153253	165,087,526–165,204,295	13.31822487	9.77E-07
LEF1-AS1	4	ENSG00000232021	108,167,525–108,304,927	13.55007746	1.27E-06
SGMS2	4	ENSG00000164023	107,823,645–107,915,047	13.45601813	1.37E-06
RPS3A	4	ENSG00000145425	151,099,624–151,104,642	13.03621022	1.40E-06
INPP4B	4	ENSG00000109452	142,023,160–142,847,432	13.35043877	1.45E-06

List of independent significant loci. Bonferroni threshold = 2.26E-06.

### 3.4 FUSION conditional analysis and colocalization results

**FIGURE 3:**
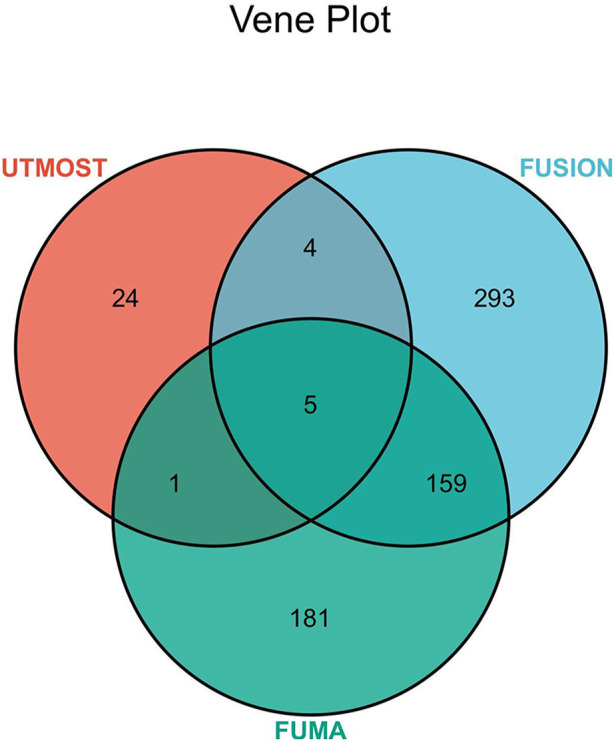
Venn diagram. FUMA identified 346 significant genes associated with hypertension, FUSION identified 461, and UTMOST cross-tissue analysis identified 34, of which 5 were common.

We identified 461 significant genes through TWAS analysis of hypertension GWAS statistical summary data and 49 tissue eQTL data from GTEx v8 using FUSION software (Supplementary Table S7). To reduce false-positive results and obtain more statistically significant association results, we combined the UTMOST cross-tissue results with the significant genes detected by FUSION and FUMA and identified five common significant genes (Figure 3; Table 2). We identified 20 significant features from 5 unique genes (*KCNK3, ENPEP, LEF1, USP38, MAPK4*) that were differentially expressed in different tissues. Among the 20 significant features, 4 were upregulated, while 16 were downregulated. The most significant feature was *KCNK3* (GTEx pancreas) (*Z* = −13.97, *p* = 2.41E-44).

**TABLE 2:** List of the significant candidate genes identified by UTMOST, FUSION and FUMA.

Genes	Tissues	Cytogenetic band	Best eQTLs	Best SNPs	Z_FUSION_	P_FUSION_	PP4	Z_FUMA_	P_FUMA_
ENSG 00000171303 (KCNK3)	Adipose Subcutaneous	2p23.3	rs11126666	rs1275985	7.01843	2.24E-12	0.291	6.8676	3.26E-12
	Brain Caudate basal ganglia		rs1275957	rs1275985	−10.88429	1.37E-27	**0.867**		
	Brain Cortex		rs1627854	rs1275985	−5.9524	2.64E-09	0.046		
	Brain Nucleus accumbens basal ganglia		rs11126666	rs1275985	−12.76961	2.42E-37	**0.845**		
	Heart Atrial Appendage		rs1275985	rs1275985	−1.35E+01	1.31E-41	**0.994**		
	Nerve Tibial		rs828259	rs1275985	6.54181	6.08E-11	0.522		
	Pancreas		rs11126666	rs1275985	−13.969	2.41E-44	0.003		
	Skin Not Sun Exposed Suprapubic		rs3806518	rs1275985	7.86772	3.61E-15	0.716		
	Cells Cultured fibroblasts	4q25	rs6533524	rs2087160	−7.2716	3.55E-13	0.13	7.8268	2.50E-15
ENSG 00000138792(ENPEP)	Colon Sigmoid		rs6818198	rs2087160	−5.8337	5.42E-09	**0.992**		
	Esophagus Gastroesophageal Junction		rs6818198	rs2087160	−6.34821	2.18E-10	**0.997**		
	Esophagus Mucosa		rs1879057	rs2087160	−5.8425	5.14E-09	**0.997**		
	Pituitary		rs2087160	rs2087160	−6.32253	2.57E-10	**0.995**		
	Spleen		rs6818198	rs2087160	−6.318	2.65E-10	**0.914**		
ENSG 00000138795 (LEF1)	Thyroid	4q25	rs4613638	rs7676998	5.74E+00	9.20E-09	**0.92**	6.0441	7.51E-10
ENSG 00000170185 (USP38)	Adipose Visceral Omentum	4q31.21	rs4337690	rs4132266	−6.426	1.31E-10	**0.982**	6.3638	9.84E-11
	Nerve Tibial		rs7670439	rs4132266	−6.426811	1.30E-10	**0.927**		
	Testis		rs11100775	rs4132266	−6.454256	1.09E-10	**0.948**		
	Whole Blood		rs7670439	rs4132266	−6.43455	1.24E-10	**0.986**		
ENSG 00000141639 (MAPK4)	Heart Atrial Appendage	18q21.1-q21.2	rs12604601	rs4599004	−5.2742	1.33E-07	**0.851**	6.2341	2.27E-10

UTMOST, FUSION, and FUMA, were used to identify genes associated with essential hypertension. And 1,000 Genomes Phase 3 (Europe, N = 489) data were used as the LD, reference panel; Z_FUSION_, is the Z-score of FUSION, result; P_FUSION_, is the *p*-value of FUSION, result; PP4 is the Bayesian colocalization posterior probalility 4; Z_FUMA_, is the Z-score of FUMA, result; P_FUSION_, is the *p*-value of FUSION, result; PP4, shared eQTL, and GWAS, associations. Bold represents PP4 > 75%.

**FIGURE 4:**
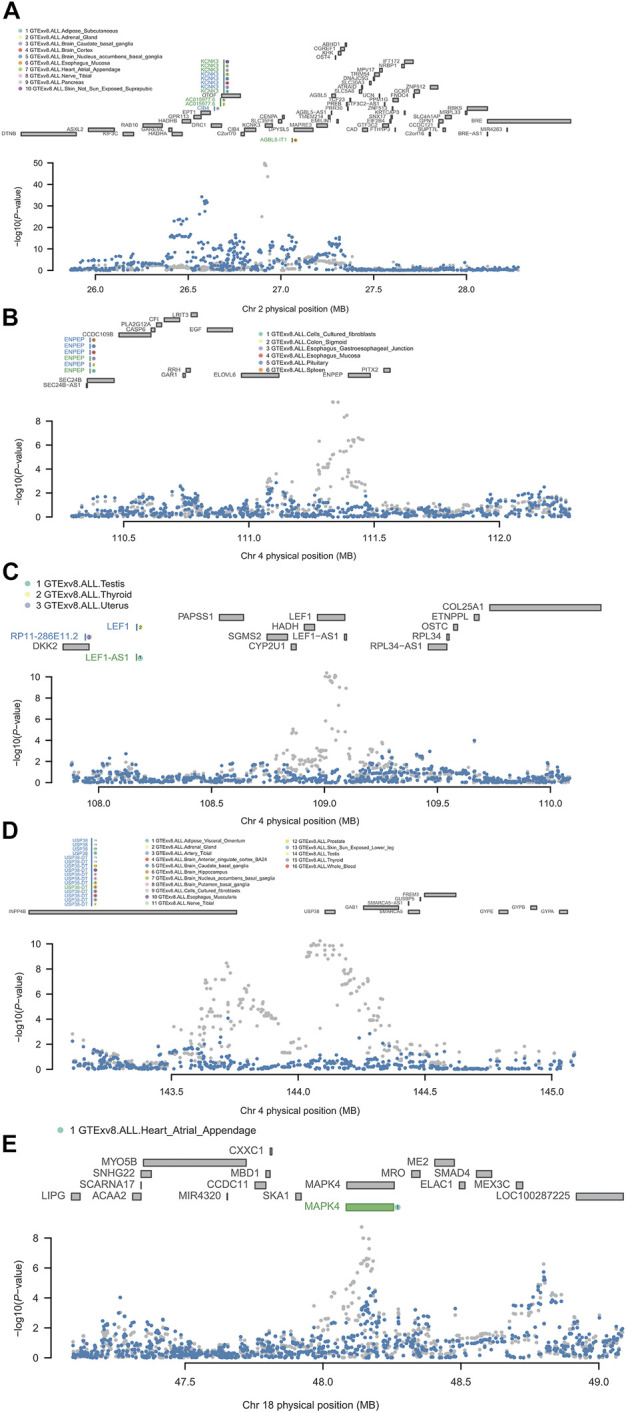
Regional association of TWAS hits. **(A)** Chromosome 2 regional association plot. **(B)** Chromosome 4 regional association plot (part 1). **(C)** Chromosome 4 regional association plot (part 2). **(D)** Chromosome 4 regional association plot (part 3). **(E)** Chromosome 18 regional association plot. The marginally associated TWAS genes are shown in blue, and the conditionally significant genes are shown in green. The bottom panel shows a regional Manhattan plot of the GWAS data before (gray) and after (blue) conditioning on the green genes’ predicted expression.

*KCNK3* is located on chromosome 2p23.3. The results of FUSION showed that it was significant in the adipose subcutaneous, brain caudate basal ganglia, brain cortex, brain nucleus accumbens basal ganglia, heart atrial appendage, nerve tibial, pancreas, skin not sun-exposed suprapubic. The results of the conditional analysis showed that the *KCNK3* gene was able to explain all the signals at this locus (rs1275985 lead SNP_GWAS_ *p* = 1.40E-50, conditioned on *KCNK3* lead SNP_GWAS_ *p* = 1) (Figure 4A). Bayesian colocalization results showed that the gene was greater than 75% of PP4 in three tissues: brain caudate basal ganglia, brain nucleus accumbens basal ganglia, and heart atrial appendage (Table 2).

*ENPEP* is located on chromosome 4q25, the results of FUSION showed that it was significant in the cells cultured fibroblasts, colon sigmoid, esophagus gastroesophageal junction, esophagus mucosa, pituitary, and spleen. It was observed that *ENPEP* explained all of the signals at its loci (rs2087160 lead SNP_GWAS_ *p* = 2.50E-10, conditioned on ENPEP lead SNP_GWAS_ *p* = 1) (Figure 4B). Bayesian colocalization resulting from *ENPEP* showed that PP4 was greater than 75% in all five tissue (Table 2), except for cells cultured fibroblasts.

*LEF1* is also located on chromosome 4q25 and the FUSION results revealed that it was only significant in thyroid tissue. It was found that *LEF1* explained all of the signals at its loci (rs7676998 lead SNP_GWAS_ *p* = 4.20E-11, conditioned on *LEF1* lead SNP_GWAS_ *p* = 1) (Figure 4C). Bayesian colocalization results showed a PP4 of 0.92 (Table 2).

*USP38* is located on chromosome 4q31.21, the results of FUSION showed its significance in the adipose visceral omentum, nerve tibial, testis, and whole blood. It was found that *USP38* explained a portion of the signal at its loci (rs4132266 lead SNP_GWAS_ *p* = 5.70E-11, conditioned on *USP38* lead SNP_GWAS_ *p* = 0.14) (Figure 4D). The Bayesian colocalization results for PP4 were greater than 75% in all four tissues (Table 2).

*MAPK4* is located on chromosome 18q21.1-q21.2 and the FUSION results illustrated that it was only significant in heart atrial appendage. It was observed that *MAPK4* only explained the weak signal at its loci (rs4599004 lead SNP_GWAS_ *p* = 1.80E-09, conditioned on *MAPK4* lead SNP_GWAS_ *p* = 0.00063) (Figure 4E). Bayesian colocalization results show a PP4 of 0.85 (Table 2).

### 3.5 SMR validation results

To verify whether there is a causal relationship between these five genes and essential hypertension, we validated the GWAS data for hypertension with the GTEx v8 data in SMR. We found that the expression of 3 genes in 10 tissues was significantly associated with hypertension (*P*_SMR_ < 0.05/number of probes), and there was no significant heterogeneity underlying the eQTL signals (*P*_HEIDI_ > 0.05) (Table 3). *LEF1* and *MAPK4* were not found to be significant in the validation of Mendelian randomization-based summary data from 49 tissues. Three features among ten tissues were downregulated, suggesting a protective effect of these three genes for essential hypertension (Table 3). The gene *ENPEP* had been found to be associated with hypertension in a meta-analysis (Surendran et al., 2016). KCNK3 was also identified to be associated with blood pressure in a genome-wide association study (Kato et al., 2015). However, these three genes were not reported in one of the largest GWAS studies on hypertension by Evangelou et al., 2018.

**TABLE 3:** SMR validates the association of 5 genes in different tissues.

Genes	Tissues	P_SMR_	P_HEIDI_	Beta
ENSG00000171303 (KCNK3)	Pancreas	8.73E-08	4.13E-01	−0.272246
	Adipose Visceral Omentum	1.45E-08	1.41E-01	−0.176958
	Artery Tibial	1.21E-07	1.91E-01	−0.291857
	Cells Cultured fibroblasts	6.34E-09	4.40E-01	−0.0547525
ENSG00000138792 (ENPEP)	Esophagus Mucosa	6.98E-07	3.40E-01	−0.123315
	Heart Atrial Appendage	8.71E-07	1.49E-01	−0.181733
	Heart Left Ventricle	2.01E-07	1.53E-01	−0.194325
	Skin Sun Exposed Lower leg	1.01E-07	2.78E-01	−0.220976
	Thyroid	2.58E-06	2.94E-01	−0.215926
ENSG00000170185 (USP38)	Whole Blood	3.04E-08	5.17E-02	−0.184669

SMR, summary data-based Mendelian randomization; HEIDI, heterogeneity in dependent instruments.

## 4 Discussion

As blood pressure is regulated by a complex network of renal, neurological, cardiac, vascular, and endocrine mechanisms under the influence of genetic and environmental factors, hypertension is likewise a multifactorial and complex trait (Padmanabhan and Dominiczak, 2021). The rapid development of genome-wide association study has identified over 1,477 common SNPs associated with blood pressure traits (Padmanabhan and Joe, 2017; Evangelou et al., 2018; Giri et al., 2019). A systematic evaluation identified 62 genes associated with essential hypertension, and after being screened according to a series of inclusion and exclusion criteria, only 21 genes were matched (Manosroi and Williams, 2019). Determining which genes are the key genes for polygenic hypertension has been an ongoing quest for researchers working on hypertension.

In our study, different methods were used to combine the essential hypertension GWAS summary statistics from European samples with eQTL data from GTEx v8. FUMA identified 346 significant genes associated with hypertension, FUSION identified 461, and UTMOST cross-tissue analysis identified 34, five of which were common. Three (*KCNK3*, *ENPEP,* and *USP38*) of these five genes (*KCNK3*, *ENPEP*, *USP38*, *LEF1,* and *MAPK4*) were simultaneously validated by SMR. Results obtained by UTMOST single-tissue analysis and FUSION software analysis showed that significant genes were detected in each of the 49 tissues of GTEx v8. This result further validates that hypertension is a disease that involving all systems of the body.

The protein encoded by *KCNK3* is sensitive to changes in extracellular pH (also named acid-sensitive potassium channel protein TASK-1). TASK-1 is expressed in a variety of cells, such as carotid vesicles, lung smooth muscle cells, motor neurons, and adrenal glomerulosa cells (Olschewski et al., 2017). It modulates muscle contraction and promotes arterial relaxation through the resting membrane potential of human pulmonary artery smooth muscle cells (Ma et al., 2013). Several studies have shown that *KCNK3* is closely associated with pulmonary arterial hypertension (Ma et al., 2013; Antigny et al., 2016; Southgate et al., 2020). A genome-wide association study identified *KCNK3* as a genetic locus that affected blood pressure (Kato et al., 2015). Animal studies have demonstrated that aldosterone is mildly elevated in TASK-1 deficient mice, causing hypertension (Manichaikul et al., 2016). It is well known that obesity is a risk factor for hypertension (Jones et al., 2012). A recent study found that *KCNK3* antagonized norepinephrine-induced membrane depolarization by promoting potassium efflux in brown adipocytes, inhibiting adrenergic signaling and thereby attenuating lipolysis and thermogenic respiration (Chen et al., 2017). Whether there is an association between *KCNK3* in the regulation of obesity and hypertension remains to be further investigated.

The *ENPEP* encodes a glutamyl aminopeptidase, also named aminopeptidase A (APA), which converts angiotensin Ⅱ (Ang Ⅱ) to angiotensin Ⅲ (Ang Ⅲ). Ang Ⅱ activates angiotensin 1 (AT1) receptors causing vasoconstriction, while Ang Ⅲ activates angiotensin 2 (AT2) receptors causing vasodilation (Te Riet et al., 2015). APA is expressed in several tissues, especially in the small intestine, kidney, duodenum, placenta, and endometrium in high amounts (Fagerberg et al., 2014). *ENPEP* was found to be associated with hypertension in a genome-wide association study in humans (Surendran et al., 2016). *ENPEP* knockout mice, infused with low doses of angiotensin Ⅱ, showed a significant increase in blood pressure (Mitsui et al., 2003). In the circulatory system, Ang Ⅲ is an AT2 receptor agonist, however, in the brain, it is an AT1 receptor agonist (O'Connor et al., 2022). In brain tissue, therefore, APA is capable of causing an increase in blood pressure. An APA inhibitor with high brain tissue selectivity--EC33, blocks the activity of APA in the brain, thereby lowering blood pressure (Llorens-Cortes and Touyz, 2020).

The *USP38*-encoded protein is involved in protein deubiquitination in the cytoplasmic matrix and nucleus. It has been found that *USP38* is a novel histone deubiquitinating enzyme that acts in concert with the histone H3K4-specific demethylase KDM5B to orchestrate the inflammatory response (Zhao et al., 2020). *USP38* deficiency-mediated inhibition of H3K4me3 demethylation enhances the inflammatory response and there is an interaction between histone ubiquitination and methylation through the USP38/KDM4B axis in gene transcriptional regulation and the inflammatory response (Zhao et al., 2020). The expression of deubiquitinases in vascular cells is important for maintaining vascular physiology and homeostasis, and it has been shown that *USP20* and *USP33* can be involved in the regulation of blood pressure by affecting the transport and stabilization of the β2AR (Wang et al., 2020). It has been shown that *USP2-45* and *USP8* can reverse the binding of the E3 ubiquitin protein ligase Nedd4-2 to epithelial Na^+^ channels, which in turn affects Na^+^ transport and uptake, which are closely associated with hypertension (Zhou et al., 2013). *USP38* knockout mice showed more severe signs of inflammatory response in acute colitis and lung injury induced by endotoxic shock (Zhao et al., 2020). Current research has linked *USP38* to the development of many diseases such as asthma (Hirota et al., 2011), pulmonary fibrosis (Yi et al., 2022), and colorectal cancer (Zhan et al., 2020). Inflammation plays an important role in the pathogenesis of hypertension and it has been suggested that the combination of impaired urinary sodium relationships, vasorelaxation, and increased sympathetic activity caused by inflammation leads to hypertension (Rodriguez-Iturbe et al., 2017). As a deubiquitinating enzyme, *USP38* plays an important role in the regulation of inflammation and this family of deubiquitinating enzymes has been found to be closely associated with the development of hypertension, so whether there is a direct association between *USP38* and hypertension needs to be further investigated.

In summary, our study identified three key genes in essential hypertension: *KCNK3, ENPEP,* and *USP38*. Among them, *ENPEP* is closely associated with hypertension, and drugs targeting this gene have been investigated. In previous GWAS studies on blood pressure regulation, the association of *ENPEP* and *KCNK3* with hypertension has been established, and the association between *USP38* and blood pressure regulation still needs further validation (Evangelou et al., 2018). Our research has certain limitations: no follow-up functional trial validation was done. The sample only included European populations, and therefore it was not possible to extend the results of the trial to the whole human population. Our study is innovative in that TWAS explores associations with traits at the genetic level, allowing more precise targeting of genes than previous SNP studies, however, no TWAS studies have been published on essential hypertension and this study adds to that. We look forward tousing large samples of different ethnic groups, multiple TWAS methods, and in-depth functional tests, to validate key genes in essential hypertension in future studies.

## Data Availability

The original contributions presented in the study are included in the article/Supplementary Material, further inquiries can be directed to the corresponding authors.

## Supplementary material

SUPPLEMENTARY TABLE S1 FUMA identified 173 genomic risk loci associated with essential hypertension.

SUPPLEMENTARY TABLE S2 FUMA identified 248 lead SNPs.

SUPPLEMENTARY TABLE S3 FUMA identified 662 independent significant SNPs.

SUPPLEMENTARY TABLE S4 FUMA annotation of candidate SNPs.

SUPPLEMENTARY TABLE S5 MAGMA gene-based test identified 346 significant genes.

SUPPLEMENTARY TABLE S6 The top 10 gene-set of MAGMA gene-set analysis.

SUPPLEMENTARY TABLE S7 UTMOST single-tissue analysis.

SUPPLEMENTARY TABLE S8 FUSION identified 461 significant genes associated with essential hypertension.

## Abbreviations

GWAS, Genome-wide association study; TWAS, Transcriptome-wide association studies; eQTL, Expression quantitative trait loci; GTEx, Genotype-Tissues Expression Project; CVD, Cardiovascular diseases; FUMA, Functional Mapping and Annotation; UTMOST, Unified Test for Molecular SignaTures; GBJ, Generalized Berk-Jones; FUSION, Functional Summary-based Imputation; SMR, Summary data-based Mendelian Randomization.

## References

[B1] Antigny F. Hautefort A. Meloche J. Belacel-Ouari M. Manoury B. Rucker-Martin C. (2016). Potassium Channel subfamily K member 3 (KCNK3) contributes to the development of pulmonary arterial hypertension. Circulation 133 (14), 1371–1385. 10.1161/CIRCULATIONAHA.115.020951 26912814

[B2] Baranova A. Cao H. Zhang F. (2021). Unraveling risk genes of COVID-19 by multi-omics integrative analyses. Front. Med. (Lausanne) 8, 738687. 10.3389/fmed.2021.738687 34557504 PMC8452849

[B3] Chen Y. Zeng X. Huang X. Serag S. Woolf C. J. Spiegelman B. M. (2017). Crosstalk between KCNK3-mediated ion current and adrenergic signaling regulates adipose thermogenesis and obesity. Cell 171 (4), 836–848. 10.1016/j.cell.2017.09.015 28988768 PMC5679747

[B4] Consortium G. T. (2020). The GTEx Consortium atlas of genetic regulatory effects across human tissues. Science 369 (6509), 1318–1330. 10.1126/science.aaz1776 32913098 PMC7737656

[B5] Dall'Aglio L. Lewis C. M. Pain O. (2021). Delineating the genetic component of gene expression in major depression. Biol. Psychiatry 89 (6), 627–636. 10.1016/j.biopsych.2020.09.010 33279206 PMC7886308

[B6] de Leeuw C. A. Mooij J. M. Heskes T. Posthuma D. (2015). Magma: Generalized gene-set analysis of GWAS data. PLoS Comput. Biol. 11 (4), e1004219. 10.1371/journal.pcbi.1004219 25885710 PMC4401657

[B7] Evangelou E. Warren H. R. Mosen-Ansorena D. Mifsud B. Pazoki R. Gao H. (2018). Genetic analysis of over 1 million people identifies 535 new loci associated with blood pressure traits. Nat. Genet. 50 (10), 1412–1425. 10.1038/s41588-018-0205-x 30224653 PMC6284793

[B8] Fagerberg L. Hallstrom B. M. Oksvold P. Kampf C. Djureinovic D. Odeberg J. (2014). Analysis of the human tissue-specific expression by genome-wide integration of transcriptomics and antibody-based proteomics. Mol. Cell Proteomics 13 (2), 397–406. 10.1074/mcp.M113.035600 24309898 PMC3916642

[B9] Forouzanfar M. H. Liu P. Roth G. A. Ng M. Biryukov S. Marczak L. (2017). Global burden of hypertension and systolic blood pressure of at least 110 to 115 mm Hg, 1990-2015. JAMA 317 (2), 165–182. 10.1001/jama.2016.19043 28097354

[B10] Genomes Project C. Auton A. Brooks L. D. Durbin R. M. Garrison E. P. Kang H. M. (2015). A global reference for human genetic variation. Nature 526 (7571), 68–74. 10.1038/nature15393 26432245 PMC4750478

[B11] Giambartolomei C. Vukcevic D. Schadt E. E. Franke L. Hingorani A. D. Wallace C. (2014). Bayesian test for colocalisation between pairs of genetic association studies using summary statistics. PLoS Genet. 10 (5), e1004383. 10.1371/journal.pgen.1004383 24830394 PMC4022491

[B12] Giri A. Hellwege J. N. Keaton J. M. Park J. Qiu C. Warren H. R. (2019). Trans-ethnic association study of blood pressure determinants in over 750,000 individuals. Nat. Genet. 51 (1), 51–62. 10.1038/s41588-018-0303-9 30578418 PMC6365102

[B13] Gusev A. Ko A. Shi H. Bhatia G. Chung W. Penninx B. W. (2016). Integrative approaches for large-scale transcriptome-wide association studies. Nat. Genet. 48 (3), 245–252. 10.1038/ng.3506 26854917 PMC4767558

[B14] Gusev A. Lawrenson K. Lin X. Lyra P. C. Jr. Kar S. Vavra K. C. (2019). A transcriptome-wide association study of high-grade serous epithelial ovarian cancer identifies new susceptibility genes and splice variants. Nat. Genet. 51 (5), 815–823. 10.1038/s41588-019-0395-x 31043753 PMC6548545

[B15] Hirota T. Takahashi A. Kubo M. Tsunoda T. Tomita K. Doi S. (2011). Genome-wide association study identifies three new susceptibility loci for adult asthma in the Japanese population. Nat. Genet. 43 (9), 893–896. 10.1038/ng.887 21804548 PMC4310726

[B16] Hirschhorn J. N. Daly M. J. (2005). Genome-wide association studies for common diseases and complex traits. Nat. Rev. Genet. 6 (2), 95–108. 10.1038/nrg1521 15716906

[B17] Hu Y. Li M. Lu Q. Weng H. Wang J. Zekavat S. M. (2019). A statistical framework for cross-tissue transcriptome-wide association analysis. Nat. Genet. 51 (3), 568–576. 10.1038/s41588-019-0345-7 30804563 PMC6788740

[B18] Jones A. Charakida M. Falaschetti E. Hingorani A. D. Finer N. Masi S. (2012). Adipose and height growth through childhood and blood pressure status in a large prospective cohort study. Hypertension 59 (5), 919–925. 10.1161/HYPERTENSIONAHA.111.187716 22493074 PMC3428923

[B19] Kato N. Loh M. Takeuchi F. Verweij N. Wang X. Zhang W. (2015). Trans-ancestry genome-wide association study identifies 12 genetic loci influencing blood pressure and implicates a role for DNA methylation. Nat. Genet. 47 (11), 1282–1293. 10.1038/ng.3405 26390057 PMC4719169

[B20] Kucmierz J. Frak W. Mlynarska E. Franczyk B. Rysz J. (2021). Molecular interactions of arterial hypertension in its target organs. Int. J. Mol. Sci. 22 (18), 9669. 10.3390/ijms22189669 34575833 PMC8471598

[B21] Levy D. Ehret G. B. Rice K. Verwoert G. C. Launer L. J. Dehghan A. (2009). Genome-wide association study of blood pressure and hypertension. Nat. Genet. 41 (6), 677–687. 10.1038/ng.384 19430479 PMC2998712

[B22] Li X. Wang H. Zhu Y. Cao W. Song M. Wang Y. (2021). Heritability enrichment of immunoglobulin G N-glycosylation in specific tissues. Front. Immunol. 12, 741705. 10.3389/fimmu.2021.741705 34804021 PMC8595136

[B23] Liao C. Laporte A. D. Spiegelman D. Akcimen F. Joober R. Dion P. A. (2019). Transcriptome-wide association study of attention deficit hyperactivity disorder identifies associated genes and phenotypes. Nat. Commun. 10 (1), 4450. 10.1038/s41467-019-12450-9 31575856 PMC6773763

[B24] Llorens-Cortes C. Touyz R. M. (2020). Evolution of a new class of antihypertensive drugs: Targeting the brain renin-angiotensin system. Hypertension 75 (1), 6–15. 10.1161/HYPERTENSIONAHA.119.12675 31786978

[B25] Luft F. C. (2001). Twins in cardiovascular genetic research. Hypertension 37 (2), 350–356. 10.1161/01.hyp.37.2.350 11230299

[B26] Ma L. Roman-Campos D. Austin E. D. Eyries M. Sampson K. S. Soubrier F. (2013). A novel channelopathy in pulmonary arterial hypertension. N. Engl. J. Med. 369 (4), 351–361. 10.1056/NEJMoa1211097 23883380 PMC3792227

[B27] Manichaikul A. Rich S. S. Allison M. A. Guagliardo N. A. Bayliss D. A. Carey R. M. (2016). KCNK3 variants are associated with hyperaldosteronism and hypertension. Hypertension 68 (2), 356–364. 10.1161/HYPERTENSIONAHA.116.07564 27296998 PMC4945430

[B28] Manosroi W. Williams G. H. (2019). Genetics of human primary hypertension: Focus on hormonal mechanisms. Endocr. Rev. 40 (3), 825–856. 10.1210/er.2018-00071 30590482 PMC6936319

[B29] Maurano M. T. Humbert R. Rynes E. Thurman R. E. Haugen E. Wang H. (2012). Systematic localization of common disease-associated variation in regulatory DNA. Science 337 (6099), 1190–1195. 10.1126/science.1222794 22955828 PMC3771521

[B30] Mitsui T. Nomura S. Okada M. Ohno Y. Kobayashi H. Nakashima Y. (2003). Hypertension and angiotensin II hypersensitivity in aminopeptidase A-deficient mice. Mol. Med. 9 (1-2), 57–62. 10.1007/bf03402108 12765341 PMC1430374

[B31] NCD Risk Factor Collaboration (2021). World wide trends in hypertension prevalence and progress in treatment and control from 1990 to 2019: A pooled analysis of 1201 population-representative studies with 104 million participants. Lancet 398 (10304), 957–980. 10.1016/S0140-6736(21)01330-1 34450083 PMC8446938

[B32] Niiranen T. J. McCabe E. L. Larson M. G. Henglin M. Lakdawala N. K. Vasan R. S. (2017). Risk for hypertension crosses generations in the community: A multi-generational cohort study. Eur. Heart J. 38 (29), 2300–2308. 10.1093/eurheartj/ehx134 28430902 PMC6075041

[B33] Nishimura T. Saeki M. Motoi Y. Kitamura N. Mori A. Kaminuma O. (2014). Selective suppression of Th2 cell-mediated lung eosinophilic inflammation by anti-major facilitator super family domain containing 10 monoclonal antibody. Allergol. Int. 63 (1), 29–35. 10.2332/allergolint.13-OA-0635 24809373

[B34] O'Connor A. T. Haspula D. Alanazi A. Z. Clark M. A. (2022). Roles of Angiotensin III in the brain and periphery. Peptides 153, 170802. 10.1016/j.peptides.2022.170802 35489649

[B35] Olczak K. J. Taylor-Bateman V. Nicholls H. L. Traylor M. Cabrera C. P. Munroe P. B. (2021). Hypertension genetics past, present and future applications. J. Intern Med. 290 (6), 1130–1152. 10.1111/joim.13352 34166551

[B36] Olschewski A. Veale E. L. Nagy B. M. Nagaraj C. Kwapiszewska G. Antigny F. (2017). TASK-1 (KCNK3) channels in the lung: From cell biology to clinical implications. Eur. Respir. J. 50 (5), 1700754. 10.1183/13993003.00754-2017 29122916

[B37] Padmanabhan S. Dominiczak A. F. (2021). Genomics of hypertension: The road to precision medicine. Nat. Rev. Cardiol. 18 (4), 235–250. 10.1038/s41569-020-00466-4 33219353

[B38] Padmanabhan S. Joe B. (2017). Towards precision medicine for hypertension: A review of genomic, epigenomic, and microbiomic effects on blood pressure in experimental rat models and humans. Physiol. Rev. 97 (4), 1469–1528. 10.1152/physrev.00035.2016 28931564 PMC6347103

[B39] Page M. J. McKenzie J. E. Bossuyt P. M. Boutron I. Hoffmann T. C. Mulrow C. D. (2021). The PRISMA 2020 statement: An updated guideline for reporting systematic reviews. BMJ Clin. Res. ed) 372, n71. 10.1136/bmj.n71 PMC8005924 33782057

[B40] Pairo-Castineira E. Clohisey S. Klaric L. Bretherick A. D. Rawlik K. Pasko D. (2021). Genetic mechanisms of critical illness in COVID-19. Nature 591 (7848), 92–98. 10.1038/s41586-020-03065-y 33307546

[B41] Restuadi R. Steyn F. J. Kabashi E. Ngo S. T. Cheng F. F. Nabais M. F. (2022). Functional characterisation of the amyotrophic lateral sclerosis risk locus GPX3/TNIP1. Genome Med. 14 (1), 7. 10.1186/s13073-021-01006-6 35042540 PMC8767698

[B42] Rodriguez-Iturbe B. Pons H. Johnson R. J. (2017). Role of the immune system in hypertension. Physiol. Rev. 97 (3), 1127–1164. 10.1152/physrev.00031.2016 28566539 PMC6151499

[B43] Russo A. Di Gaetano C. Cugliari G. Matullo G. (2018). Advances in the genetics of hypertension: The effect of rare variants. Int. J. Mol. Sci. 19 (3), 688. 10.3390/ijms19030688 29495593 PMC5877549

[B44] Southgate L. Machado R. D. Graf S. Morrell N. W. (2020). Molecular genetic framework underlying pulmonary arterial hypertension. Nat. Rev. Cardiol. 17 (2), 85–95. 10.1038/s41569-019-0242-x 31406341

[B45] Surendran P. Drenos F. Young R. Warren H. Cook J. P. Manning A. K. (2016). Trans-ancestry meta-analyses identify rare and common variants associated with blood pressure and hypertension. Nat. Genet. 48 (10), 1151–1161. 10.1038/ng.3654 27618447 PMC5056636

[B46] Te Riet L. van Esch J. H. Roks A. J. van den Meiracker A. H. Danser A. H. (2015). Hypertension: Renin-angiotensin-aldosterone system alterations. Circ. Res. 116 (6), 960–975. 10.1161/CIRCRESAHA.116.303587 25767283

[B47] Wang B. Cai W. Ai D. Zhang X. Yao L. (2020). The role of deubiquitinases in vascular diseases. J. Cardiovasc Transl. Res. 13 (2), 131–141. 10.1007/s12265-019-09909-x 31823221

[B48] Watanabe K. Taskesen E. van Bochoven A. Posthuma D. (2017). Functional mapping and annotation of genetic associations with FUMA. Nat. Commun. 8 (1), 1826. 10.1038/s41467-017-01261-5 29184056 PMC5705698

[B49] Wellcome Trust Case Control C. (2007). Genome-wide association study of 14,000 cases of seven common diseases and 3,000 shared controls. Nature 447 (7145), 661–678. 10.1038/nature05911 17554300 PMC2719288

[B50] Willer C. J. Li Y. Abecasis G. R. (2010). Metal: Fast and efficient meta-analysis of genomewide association scans. Bioinformatics 26 (17), 2190–2191. 10.1093/bioinformatics/btq340 20616382 PMC2922887

[B51] Yi X. M. Li M. Chen Y. D. Shu H. B. Li S. (2022). Reciprocal regulation of IL-33 receptor-mediated inflammatory response and pulmonary fibrosis by TRAF6 and USP38. Proc. Natl. Acad. Sci. U. S. A. 119 (10), e2116279119. 10.1073/pnas.2116279119 35238669 PMC8917384

[B52] Zhan W. Liao X. Liu J. Tian T. Yu L. Li R. (2020). USP38 regulates the stemness and chemoresistance of human colorectal cancer via regulation of HDAC3. Oncogenesis 9 (5), 48. 10.1038/s41389-020-0234-z 32404892 PMC7220910

[B53] Zhao Z. Su Z. Liang P. Liu D. Yang S. Wu Y. (2020). USP38 couples histone ubiquitination and methylation via KDM5B to resolve inflammation. Adv. Sci. (Weinh). 7 (22), 2002680. 10.1002/advs.202002680 33240782 PMC7675183

[B54] Zhou B. Perel P. Mensah G. A. Ezzati M. (2021). Global epidemiology, health burden and effective interventions for elevated blood pressure and hypertension. Nat. Rev. Cardiol. 18 (11), 785–802. 10.1038/s41569-021-00559-8 34050340 PMC8162166

[B55] Zhou R. Tomkovicz V. R. Butler P. L. Ochoa L. A. Peterson Z. J. Snyder P. M. (2013). Ubiquitin-specific peptidase 8 (USP8) regulates endosomal trafficking of the epithelial Na+ channel. J. Biol. Chem. 288 (8), 5389–5397. 10.1074/jbc.M112.425272 23297398 PMC3581384

[B56] Zhu Z. Zhang F. Hu H. Bakshi A. Robinson M. R. Powell J. E. (2016). Integration of summary data from GWAS and eQTL studies predicts complex trait gene targets. Nat. Genet. 48 (5), 481–487. 10.1038/ng.3538 27019110

